# Hospital and Institutional News

**Published:** 1917-03-24

**Authors:** 


### HOSPITAL AND INSTITUTIONAL NEWS.

### THE DUCHESS OF CONNAUGHT.

AMONGST the tributes of respect and affection elicited by the death of Her Royal Highness the Duchess of Connaught, there will be few more sincere than those, often unspoken, which fill the hearts of so many institutional workers. The Duchess's health during recent years, and her long absence on Imperial duties in Canada, have made her appearances at our hospitals less frequent of late years than she would have wished them to be; but this cannot efface the memory of her keen sympathy with the sick and suffering, and of her enduring interest in institutional affairs. In this connection it is worth recalling a fact which seems to have escaped the notice of most of those who have compiled the memorial notices in the daily press, but deserves to be put on record. This is that the Duchess's last public appearance, on the very day before her fatal illness began, was for the purpose of formally opening the Countess of Lytton's new auxiliary hospital for wounded soldiers in Charles Street, Berkeley Square. This hospital is housed in the palatial residence of the Earl of Dartmouth, who has kindly lent it for conversion into a hospital; and is a fine example of the generosity of the nobility in lending their residences to serve the country's need, of the devoted services rendered in so many of this type of hospital by V.A.D. and other voluntary workers, and of the British national genius for adaptation and for obtaining practical results. At the opening by the Duchess of Connaught, she made one of those simple, straightforward speeches which reflected so accurately her high character and sound sense. To the Duke, and to his children, the whole hospital world tenders respectful sympathy in their bereavement.

### THE HOSTEL COMMITTEE FOR VICTIMS OF NERVE STRAIN.

SIR FREDERICK MILNER, who has done so much for various kinds of invalided sailors and soldiers, has worked hard of late in organising hostels for the treatment of the victims of nerve strain. One of these institutions is to be opened, for sufferers out of both Services, at Hampstead; and it has recently been announced that Mrs. McIntosh has offered her splendid house and grounds of Marshall's Park, Romford, to the Hostels Committee for the same purpose. The *Daily Graphic*, which is appealing for subscriptions towards the cost of this scheme of recuperative hostels, describes the system as one of curative treatment of nerve-strain due to the war, the re-equipment of patients for a fresh start in life, and country homes to complete recovery through facilities for useful and interesting occupation, more especially in the open air. Various branches of gardening, bee and fish culture, carpentry, rural wood industries, dairy and poultry farming are amongst the occupations that will be taught. These industries will, it is stated, be run on practical business lines under experts who have promised their services free of charge; and the salaried instructors will, as far as possible, be invalided officers and men. Those men who have previously had a trade will be encouraged to resume their former occupations.

### MR. PETER REID'S BEQUESTS.

ONLY a few weeks ago (THE HOSPITAL, February 17, p. 396) attention was drawn in a special memoir to the life-work of Mr. Peter Reid, the founder of the Hospital Convalescent Home, Parkwood, Swanley. Now the disposal of his property has been made known, and a princely list of benefactions is disclosed. The value of the estate is £129,683. Out of this there is bequeathed to the Hospital for Sick Children, Great Ormond Street, £20,000 to erect and help to maintain a convalescent home for the patients of the hospital, at a distance of between five and twenty miles from Charing Cross. In a communication to the press, the secretary of this hospital points out that the erection and equipment of the home will absorb practically the whole of the legacy, and that hardly anything will be left for endowment and maintenance. The following hospitals and institutions receive £500 apiece: the Hospital for Consumption and Diseases of the Chest; the London Hospital; the Metropolitan Convalescent Institution; St. Mary's Hospital; St. Thomas's Hospital; the Scottish Hospital, Crane Court, E.C.; the Westminster Hospital; and the Stock Exchange Benevolent Fund. There are some interesting bequests to officials of the Hospital Convalescent Home, Swanley: such as £300 to Ethelle Campbell, the matron; £200 to Miss Thomson, assistant matron; £100 to William Hobbs, the engineer; £200 to the nurses; and £200 to the outdoor staff. Except for a few similar legacies, including £100 each to two nurses who attended the testator, the whole of the rest of Mr. Reid's fortune is left to the institution which he founded, and with which his name will now more than ever be inseparably connected.

### ANOTHER LARGE ESTATE FOR INSTITUTIONS.

THE details of yet another large estate devoted almost wholly to institutional charities fall to be announced this week. Under the will of Mrs. Edith Harriet Scott, of Derby, some £3,000 is distributed in varying amounts among twenty-seven different charities, including £1,000 to St. George's Hospital, Hyde Park Corner, and between £3,000 and £4,000 to the National Lifeboat Institution. The total value of the estate is just over £73,000; and the residue, which must be of very considerable magnitude, is to be divided equally between Guy's Hospital, St. George's Hospital, and the Hospital for Diseases of the Heart, Soho Square. The account of the legacies as published in the press is not exhaustive, so that it is impossible to judge precisely to what extent these three fortunate institutions will benefit; but it is fairly clear that each will receive several thousand pounds under this will, which is as noteworthy in its way as that of Mr. Peter Reid.

### TUBERCULOSIS IN THE ARMY.

In the *Daily Telegraph* of the 13th inst. appears a paragraph setting forth the views on the above subject of the National Association for the Prevention of Tuberculosis. It is considered that "under existing regulations, whereby soldiers suffering from any form of tuberculous disease are forthwith discharged from the Army, it is not possible to be certain that they will avail themselves of the treatment offered in a civilian institution," or will stop there long enough. They suggest the retaining of the man in the Army for a specified maximum time, not indeed in a military institution, but while he is resident in a special hospital or sanatorium for tuberculosis. They suggest, too, that the services of officers in the R.A.M.C. who are experts in tuberculosis should be more definitely utilised in relation to the diagnosis and treatment of tuberculous soldiers. The suggestion is a good one, and the plan advised one of the many things Germany did at the outset of the war, and other countries are seeking to effect near the end of it. From the outset there was a special tuberculosis section of the German Red Cross, largely staffed by specialists. And as we mentioned the other week, Swiss doctors in Davos were given military authority over German tuberculous soldiers interned there. For sanatorium routine is not one of the "great delights" which, as Bacon said and as one sees on all hands, are a common correlative to "great dangers." In winter especially, the poor consumptive soldier—and it is hard to blame him—is attracted by a more comfortable and gayer life, and unless he meets a born physician, will exercise his power of free movement in a way unfortunate for his health. The Council states that the experts in tuberculosis now in the R.A.M.C. are "numerous." Certainly, and fortunately, there are some. But so many tuberculosis officers have been elected on grounds quite other than clinical—latterly often because engaged in posts they can leave at short notice—that the word "numerous" is erroneous.

### ATTENDANCE AT ANNUAL MEETINGS.

Colonel Perkins, chairman of the board of the Royal South Hants and Southampton Hospital, struck the right note in remarking at the recent annual meeting on the numerous attendance, which he said was larger than any he remembered. He hoped it showed increased interest in the hospital. This seems to be shown by the fact that the unavoidable increase in expenditure has been nearly equalled by a rise in income, and we have a personal interest in the position in having recommended this hospital in the past to organise its supporters in the outlying districts, from which many patients come, more carefully. Such organisation does not only mean collections, it means the drawing closer of the ties of personal interest from which support and subscriptions mainly flow. Annual meetings are often regarded as a tiresome formality, which is necessarily dull, and should be passed over as quickly as decency permits. The fact remains, however, that a hospital which can keep its annual meeting packed is never a hospital which is not abreast of its responsibilities or long without the means to shoulder them.

### REMOVAL OF CHILDREN FROM HOSPITAL CONSTRUED AS PARENTAL CRUELTY.

The satisfaction which every social worker must feel at the conviction of two parents at West Bromwich for removing their children from hospital at a time when they were not fit to be removed, may not improbably be mitigated by the amazingly lenient punishment awarded by the stipendiary for what was morally, though not legally, the murder of the two unfortunate children. The circumstances of the case are that a man earning 6s. 6d. a day (with 33s. a week brought home by his elder children) was, with his wife, prosecuted for cruelty to his younger children. One of them was scalded, admitted to hospital, and then removed by the mother a few days later in spite of the protest of the hospital authorities. The child died ten days later, and the evidence showed that if it had been properly looked after it would not have died at all. The other child died of pneumonia, and was taken through the streets to the doctor the very night before his death. For these scandalous and aggravated acts of cruelty each parent was fined the sum of 20s., or seven days' imprisonment in default. Whatever views one may hold as to the adequacy of their punishment, it is at least something that the removal of a child from hospital against the advice of the doctor can be construed as cruelty punishable by the law; though it is to be hoped that other offenders, besides those whose children actually die as a result, will be brought to justice for such gross parental negligence.

### THE FOLLY OF CLOSING A COTTAGE HOSPITAL TO-DAY.

The official reason for closing the Hayes Cottage Hospital, in Buckinghamshire, is that owing to the scarcity of nurses a matron is not obtainable. Obviously this, if not actually incorrect, is insufficient, and the truth appears to be that, owing to great industrial developments which have occurred in the parish, the management of the hospital was unequal to the occasion, and failed to secure the support which the new element should have provided. Difficulties resulted, and the management apparently took advantage of the alleged scarcity of matrons and nurses to close the hospital, and throw up the sponge. This, we may point out, is neither courageous nor sensible. The factories now established bring a population, and are accompanied by accidents which necessitate an increased, not a diminished, number of hospital beds. To close the hospital in a huff because the new factory population wishes some measure of representation and control is unpatriotic and ridiculous. If the old board rules itself out as hopeless, we trust that a committee of local residents may at once be formed, with representatives of the workers upon it, to undertake the reopening of the Hayes Cottage Hospital at the earliest date. In the face of loyal co-operation petty difficulties will vanish, and the disgrace of closing a cottage hospital at a critical moment be removed from the whole county of Buckingham.

### A "VANISHING" CLASS IN YORK.

THE finance of the York County Hospital continues to give cause for anxiety in spite of the special appeals and contributions which, with the help of legacies, have enabled it to set aside a balance of £1,500 towards meeting its accumulated deficits. The annual subscriptions—serious symptom—are lower than they have ever been since 1882. This is attributed to the absence or the disappearance of the county gentlemen. As a set-off to this the workpeople's subscriptions have risen to £2,000, and under wise encouragement may yet increase. A cathedral city like York, however, should not have to complain of a miserable supply of annual subscriptions, for it possesses the class from which they come, both within and without the city walls. Since the number of in-patients has increased by 100 over that admitted in 1915, and Mr. W. F. H. Thomson, chairman, declares that "there was not a single urgent case the admission of which was delayed," an unanswerable and stirring case can be made out for help from those who, in such a city, are well able to give annual subscriptions.

### SEA TALES AND FOOD RATIONS.

IF someone would write a volume on the part the mercantile marine has taken in the war and issue it as an appeal for the Seamen's Hospital, Greenwich, or its few kindred hospitals, a double work of value would be performed. What is wanted are the untold stories which lie behind the weekly return that states that so many (and how many) merchant vessels have arrived safe in English ports in spite of mines and submarines, and the proper perils of navigation. Such sea stories would stir the public imagination and enforce Sir Perceval Nairne's assurance that without curtailing the number of beds for the men in the merchant service, the accommodation has been increased, and nearly 200 sick and wounded naval ratings have been under treatment regularly. The food supply at the Dreadnought Hospital has been found to work out, it is said, at almost exactly Lord Devonport's figures. The hospital claims to save a considerable supply of sugar and meat for the country, but Captain A. W. Clarke has declared that it cannot come down to the Devonport ration for bread. The connection between our food supply and the mercantile marine is obvious, and is happily emphasised by the work done at the Seamen's Hospital in both respects.

### THE PUBLIC PERFORMANCE OF "DAMAGED GOODS."

IT is a remarkable fact, in which THE HOSPITAL may claim a personal pleasure, that the English version of M. Brieux's play called "Damaged Goods" was publicly performed and licensed by the Court censor for the first time at the St. Martin's Theatre last Saturday. The society which is responsible for the production of the play includes men and women doctors, social workers and members of Parliament who know what a powerful weapon it has already proved in France and America against that ignorance which is such a potent factor in the spread of venereal disease. We hope that the society in question will patriotically add to its repertory an equally powerful and more human play on the same theme, and with the same object, by an English playwright—namely, Mr. Frank G. Layton's "Philip's Wife," which was published three years ago by Mr. A. C. Fifield. As far back as May 16, 1914, THE HOSPITAL published an article, entitled "The Facts of Syphilis on the Stage," in which the two plays mentioned were commended to the attention of all persons anxious to educate the public in a knowledge of the facts. The reason why the theatre is the proper place for such instruction was explained, and the plays were analysed and contrasted. At that time evidence was being given before the Royal Commission, and we are glad to see that the argument we put forward in THE HOSPITAL has proved its efficacy within three years of the time that we published it.

### A RETROGRADE STEP AT MERTHYR.

WE regret to announce that the executive committee of the Merthyr General Hospital has decided to close eleven beds as a consequence of the serious financial position of the institution. It is further stated that the closing of eleven beds "means approximately a saving of about £1,100 per annum," but if this suspiciously vague phrase is based on the assumption that the closing of so many beds means the saving of the cost per bed of each bed closed, it is a transparent inaccuracy which will deceive no hospital manager. The expense of administration remains almost constant whether a hospital has eleven beds more or less. The old fallacy that to close beds saves correspondingly in expenditure dies hard, but in war time, when every available bed is required, to put it forward is specious and unpatriotic. To learn in due course that £1,100 has not been saved, and that there is still a deficiency, will be no consolation, nor will it do more than exasperate the public. The proper course is for the district to insist on the immediate reopening of the beds, and to back the demand by a promise of effort to meet the deficiency. There is no real difficulty in this, and we hope that the present retrograde step will be retrieved immediately.

### THE MAINTENANCE OF SANATORIUM BEDS.

BARRASFORD SANATORIUM, Northumberland, an institution which has done much useful work and been fortunate in its medical staff, is one of those sanatoria the beds in which are largely reserved and paid for by different public bodies and industries. In the face of the rise in cost of living, the committee has been led to revise the rate at which beds are thus reserved, and has raised the charges by two adjustments to £150 per annum for each patient. What is more, the contracting bodies have agreed to this, but the committee, in spite of a deficit of £141, which even the increased rate has not prevented, hopes when normal conditions return to revert, in part at least, to the former smaller rate. How far prices may go down after the war no one can say, but the fact is worth recording that at Barrasford at least the cost per bed has now risen to over £150 per annum.

### THE UNPROVIDED CONSUMPTIVE IN WALES.

At a recent meeting of the Guardians the presence of tuberculous patients in the Merthyr Infirmary was under discussion. Twenty-seven in all, the chairman, Mr. T. T. Jenkins, suggested they should be sent for examination to the clinic and, if possible, admitted at the Mardy Tuberculosis Hospital. Another speaker stated that since the Merthyr Corporation and the Glamorgan County Council were contributors to the Welsh Memorial Association, it was unreasonable to expect the ratepayers to pay again, through the Guardians, for the upkeep of consumptives. It was then affirmed that all the Association's sanatoria are full, and that it has a very long waiting-list. If this be so, we fear that the ratepayers will either have to make through their representatives much larger contributions to the Welsh Memorial Association, or that they will have through the Guardians to charge themselves with the upkeep of consumptives in the wards of their infirmaries. There is a good deal to be said for the latter plan if the phthisis wards in the infirmaries are up to date and skilled medical supervision is obtainable.

### AN EXTRAORDINARY DECISION.

The respect in which the institutional world and the medical profession hold the House of Commons is not likely to be increased by an extraordinary decision of the House of Commons Tribunal to grant exemption to an unqualified practitioner of military age. The man in question devotes himself, according to the statements of his representative, to the cure of eye diseases, which he brings about by the use of lenses instead of surgical operations! In support of the application a somewhat vehement Admiral was called, who profoundly believes in the applicant's powers; and several witnesses stated that they were nearly blind until fitted with glasses by him. A list of persons alleged to have been patients of the applicant seems also to have been admitted as evidence before this extraordinary Tribunal, though the newspaper reports do not say whether the list was sworn to. No trouble whatever seems to have been taken to test the value of the evidence given, which was doubtless all in good faith but probably quite unreliable. The grateful Admiral, for example, was convinced that many people are absolutely dependent on the unqualified man for their sight, and that no other person can restore it. This grotesque statement, and the equally nonsensical statements of the counsel who supported the application, are pitiable enough, regarded merely as delusions, which anyone who knows anything whatever about eye diseases must know that they are. But to have these solemn absurdities "swallowed whole" by a House of Commons Tribunal is too much for one's gravity, more especially in conjunction with the Chairman's description of the applicant as "a sort of Mr. Barker for the eye," and his sapient remark that "we will make inquiries of Sir Arthur Pearson as to the possible utilisation of this man in National Service at home." The name of this egregious Chairman is Mr. Maclean, whose credulity is truly tragic in one who helps to make our laws.

### THE CENTENARY OF A WELSH HOSPITAL.

The Swansea Hospital is approaching its centenary year, and doubtless the anniversary will be marked in some outstanding and suitable manner. The board has been able to induce Mr. Roger Beck, a gentleman who combines in a remarkable degree the heart of the philanthropist with the mind of a successful man of business, to accept office in this auspicious year. The hospital has been fortunate in its chairmen right from the start, but a better choice than that which has been made on this occasion would be almost impossible. Mr. Beck has already given a hint of his desire to make notable his tenure of office. In accepting the chairmanship he declared that the Swansea Hospital can and shall retain its position as the western centre for Wales, adding that it must be extended so that there will not be such large waiting-lists. The centenary celebration is likely to include a campaign for freeing the institution from its last penny of debt. Mr. Roger Beck, it may be added, has recently set up in the hospital a memorial tablet to his brother, Dr. Marcus Beck, the surgeon.

### CATHETERISATION OF MALE PATIENTS.

The Appeal Tribunal for the County of London at a sitting last week expressed the opinion that there is no office in respect of the nursing of a male patient that a female nurse cannot perform, and the military representative said that he had had personal experience of the passing of a catheter on a male patient by a female nurse. The occasion was the appeal against the decision of a local Tribunal which directed the immediate joining up of a male nurse, one of two left out of a staff of sixteen in the service of a large special hospital. The Appeal Tribunal was informed that in this country it is hospital practice that the catheterisation of male patients is carried out either by the resident medical staff or by properly trained male nurses, and expressed incredulity and astonishment. It would be interesting to learn if female nurses are being instructed in the work in question, especially in view of the shortage of resident medical officers and the consequent increased duties that fall to their share. In our view catheterisation is a procedure which very few male (or female) nurses are sufficiently adept in aseptic technique to be entrusted with: the dangers of septic infection of the bladder are very great when it is attempted by any but a qualified practitioner.

